# Effects of Walking on Coronary Heart Disease in Elderly Men with Diabetes

**DOI:** 10.3390/geriatrics3020021

**Published:** 2018-04-19

**Authors:** Chieko Kimata, Bradley Willcox, Beatriz L. Rodriguez

**Affiliations:** 1 Patient Safety & Quality Services, Hawai’i Pacific Health, Honolulu, HI 96813, USA; 2 Department of Geriatric Medicine, John A. Burns School of Medicine, University of Hawai’i at Mānoa, Honolulu, HI 96817, USA; willcox@hawaii.edu (B.W.); brodrigu@hawaii.edu (B.R.); 3 Escuela de Medicina, Tecnologico de Monterrey, Monterrey, NL 64710, Mexico

**Keywords:** coronary heart disease, diabetes, walking, Honolulu Heart Program, elderly men

## Abstract

Previous studies have shown that walking is associated with increased longevity and a reduced risk of cardiovascular and age-related diseases. Whether walking benefits individuals with diabetes who are at high risk of coronary heart disease (CHD) remains to be determined. The objective of this study is to examine the association between walking and risk of CHD among elderly men with and without diabetes. Walking data was assessed in 2732 men aged 71 to 93 years participating in the Honolulu Heart Program from 1991–1993. Study participants were initially without disabilities and free of prevalent CHD. Men were then followed for incident CHD for up to 7 years. For men with diabetes who walked <0.25 miles/day, the age-adjusted incidence of CHD was significantly higher than in men without diabetes (27.1 vs. 12.7/1000 person years, *p* = 0.026). In contrast when distance walked was >1.5 miles/day, incidence of CHD was similar in men with and without diabetes (12.2 vs. 9.1/1000 person-years, *p* = 0.46). While risk of CHD declined significantly with increasing walking distance in men with diabetes after age and risk factor adjustment (*p* = 0.043, *p* = 0.025), associations in those without diabetes were weaker (*p* = 0.070, *p* = 0.10). These findings suggest that among elderly men with diabetes who are capable of physical activity, walking reduces CHD risk to levels similar to when diabetes is absent. Walking is an easy, safe and accessible form of physical activity that may have marked health benefits for elderly men with diabetes.

## 1. Introduction

Evidence suggests that walking is associated with increased longevity and a reduced risk of cardiovascular and age-related diseases [1,2,3,4]. Other benefits of walking include lowered blood pressure, reduced body weight and improved glucose and lipid metabolism [5]. Some research also shows exercise brings health benefits to people with diabetes [6,7,8]. Diabetes greatly increases the risk of future cardiovascular morbidity and mortality [9,10,11,12]. Whether walking provides benefits for elderly diabetic individuals who may possess a high risk of coronary heart disease (CHD) remains to be determined. The purpose of this research is to examine the association between walking and CHD incidence in elderly men with diabetes. Findings are based on longitudinal follow-up of a population-based sample of Japanese-American men aged 71 to 93 years who were enrolled in the Honolulu Heart Program without prevalent CHD.

## 2. Materials and Methods

### 2.1. Study Population

The Honolulu Heart Program, established in 1965, is a prospective study of cardiovascular disease and stroke [13,14,15,16]. The target population was comprised of 8006 Japanese-American men aged 45–68 at baseline examination and living on the island of Oahu, Hawai‘i. This analysis included survivors of the original cohort who participated in the 1991–1993 examination, when men were 71–93 years old. They were followed until 1998 for the development of incident CHD. Only men who were capable of physical activity were considered for this study. Men were considered to be capable of physical activity if they participated in a baseline clinical examination at the Kuakini Medical Center and reported they could undertake more than 1 h of slight, moderate or heavy activity in a typical 24-h period [4]. The sample size was 2637 after excluding those who were not eligible and those who did not have information on walking distance or diabetes status.

### 2.2. Measures

The average walking distance per day was asked of the participants at baseline examination [16]. Walking distance was treated as both a continuous variable and as a categorical variable. Categorically, variables were divided into three groups: less than 0.25 miles/day, between 0.25 and 1.5 miles/day and over 1.5 miles/day [4]. 

CHD events were acquired by a comprehensive surveillance of hospital discharge registers, health certificates, autopsy records and by repeating examinations during follow-ups. CHD was defined as unequivocal findings through hospital surveillance of nonfatal myocardial infarction with ECG or cardiac enzyme evidence, coronary death and sudden death within an hour that could not be attributed to another cause [4]. Medical history or a fasting glucose greater than 125 mg/dL was used to determine diabetes at the 1991–1993 examination [11]. Additional data on cardiovascular disease (CVD) risk factors in this cohort have been previously published [4,11,12,13,14,15,16].

### 2.3. Statistical Analysis

An age-adjusted incidence rate per 1000 (person-years) was calculated for CHD outcome by direct method of standardization using the Honolulu Heart Program population in the 1991–1993 examination. To describe the difference in incidence rates between men with diabetes and without diabetes, the cox model was used. To identify possible confounding variables between diabetic status and walking distance categories, the age-adjusted mean level of individual factors was calculated across the ranges of walking distances with the ANCOVA method using linear and logistic regression models [17]. To examine the independent effects of walking distance, possible confounding variables were adjusted in separate models. These confounders included age, body mass index (BMI), systolic blood pressure (SBP), fasting glucose, total and high-density lipoprotein (HDL) cholesterol, alcohol consumption and smoking [12,18].

Proportional hazard regression models were used to estimate the Relative Risk (RR) for the higher two levels of walking distance using the lowest level (<0.25 miles/day) as a reference group for men with and without diabetes separately. A test for trend was done using walking distance as a continuous variable to examine a dose-response relationship between walking and CHD risk. The statistical analysis was performed using the SAS statistical software version 9.3 (SAS Institute, Cary, NC, USA). A *p*-value of <0.05 was considered statistically significant.

## 3. Results

Among the 2637 men capable of physical activity and free of CHD, 538 (20%) had diabetes and 2109 (80%) did not have diabetes. The study average walking distance was 1.2 ± 1.4 miles. Among men with diabetes, 30% walked <0.25 miles/day, 41% walked 0.25 to 1.5 miles/day and 29% walked >1.5 miles/day, while 30%, 39% and 31% of men without diabetes walked these amounts, respectively. The number of men with diabetes and without diabetes who developed new CHD events was 61 (20.1 per 1000 person-years) and 149 (12.0 per 1000 person-years), respectively (Table 1).

**Figure 1:**
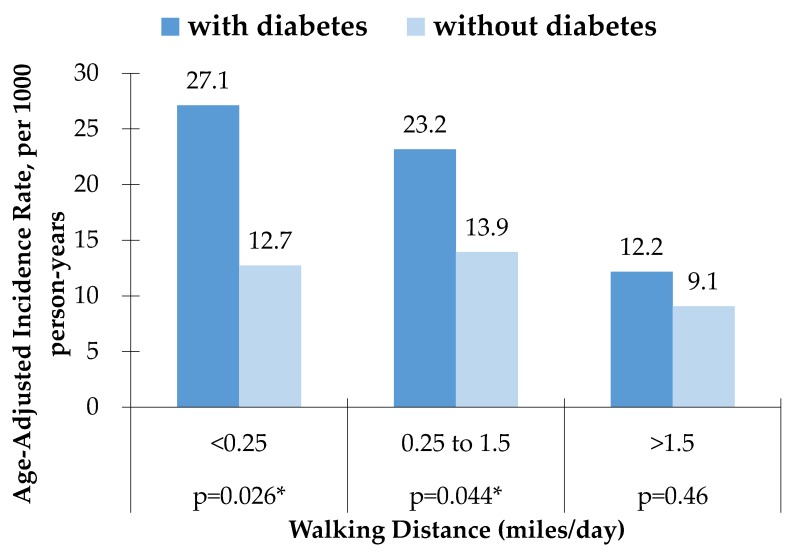
Age-Adjusted Incidence rate, per 1000 person-years of CHD among men with diabetes and without diabetes. * Significant difference between men with diabetes and without diabetes.

The age-adjusted CHD incidence rate per 1000 person-years was higher for men with diabetes than men without diabetes in all groups of walking distances, with significant differences noted in subjects who walked <0.25 miles/day (*p* = 0.026) and those who walked from 0.25 to 1.5 miles/day (*p* = 0.044). Conversely, the age-adjusted CHD incidence rate was similar among men with diabetes and without diabetes for those who reported walking >1.5 miles per day (Table 1 and Figure 1).

Table 2 shows other risk factors associated with walking distance at baseline examination. Younger men were more likely to walk longer distances in both diabetic and non-diabetic categories. Among men without diabetes, those who walked <0.25 miles/day had significantly lower SBP than those who walked >1.5 miles/day. Men who walked <0.25 miles/day were more likely to be smokers than those who walked >1.5 miles/day (*p* < 0.05). There were no associations observed between walking distance and body mass index, fasting glucose, total and HDL cholesterol, or alcohol intake.

After adjustments for age, BMI, SBP, fasting glucose, total and HDL cholesterol, alcohol consumption and smoking, the RR of CHD among men with diabetes was 0.45 (95% CI: 0.22–0.93) for those who walked >1.5 miles/day compared to men who walked <0.25 miles/day and 0.81 (95% CI: 0.47–1.41) in men who walked 0.25 to 1.5 miles/day compared to men who walked <0.25 miles/day. Among men without diabetes, the RR of CHD was 0.74 (95% CI: 0.47–1.17) in men who walked >1.5 miles/day compared to men who walked <0.25 miles/day and 1.16 (95% CI: 0.78–1.72) in men who walked 0.25 to 1.5 miles/day compared to men who walked <0.25 miles/day. There was no statistically significant interaction of diabetes with walking distance. Among men with diabetes, there was an inverse trend of walking distance and CHD (*p* = 0.043 with age-adjustment, *p* = 0.025 with risk factor-adjustment). This trend missed the significance level among those without diabetes (*p* = 0.070 with age-adjustment and *p* = 0.10 with risk factor-adjustment). In this analysis, walking distance was used as a continuous variable (Table 3).

## 4. Discussion

According to a former study by the Honolulu Heart Program, walking was associated with a reduced risk of CHD in elderly men [4]. However, the effect of walking among men with diabetes and without diabetes was not examined in the previous investigation. This study considered CHD incidence among men with diabetes and without diabetes separately and the follow-up period was longer than the former study (1 to 3 years versus 5 to 7 years). Additionally, only men who were capable of physical activity were considered. 

Having diabetes is known to increase the risk of CHD. This research suggests there is a protective effect of walking distance on CHD. This effect is stronger among men with diabetes than men without diabetes the longer the daily average walking distance. Men with diabetes who walked >1.5 miles/day had reduced levels of risk, similar to men without diabetes. Although in need of confirmation, this study suggests regularly walking 1.5 miles per day or more could be an effective way of reducing the risk of CHD in elderly men with diabetes.

One of the strengths of this paper is the quality of the data. The Honolulu Heart Program is one of the largest studies of CVD in a minority population in the United States. Comprehensive examinations were conducted with quality control and standardized procedures [19]. The Honolulu Heart Program also provides high quality longitudinal data that enables researchers to obtain prospective CHD outcomes from a comprehensive surveillance system. This includes review of examination results, all hospital discharge summaries, autopsy reports and death certificates [20]. Furthermore, approximately 80% of cohort survivors participated in the 1991–1993 examination. The study was also conducted on the island of Oahu and because of its’ isolation, the migration rate was very low. Only 5 participants were lost to follow-up of the original 8006 subjects between 1965 and 1993. Few patients left Oahu to receive medical care.

A limitation this study did not consider is the intensity and duration of walking. However, another Honolulu Heart Program study analyzed the time to walk 10 feet (intensity) and time spent in slight activities (duration in activities similar to casual walking) [16]. Another study also commented that there was a relationship between strength and power to walking behaviors in older adults [21]. Strength and duration of walking may be related to the amount of benefits from walking. This study also did not consider different forms of physical activities such as running, biking, swimming and so forth, as the participants were 71 to 93 years old and only a small percentage of men were engaged in heavy activities. However, many studies in younger populations showed a reduction in cardiovascular risks with these type of physical activities [22,23,24,25]. This investigation only included Japanese-American males. It is unclear if the findings of a sample of Japanese-American men apply to other population segments. In general however, risk factor associations with several disease endpoints in the sample from Hawai‘i closely agree with findings reported elsewhere, including relationships with CHD, dementia and Parkinson’s disease [16,26,27,28,29,30]. In direct comparison with the Framingham Study, similar risk factor effects on the incidence of stroke were observed [26]. Additional research among elderly women would lend support to our analysis, although findings are likely to be similar to those reported here [31,32]. In this analysis, only baseline data of walking distance and biological characteristics were considered, therefore, beneficial effects on biological mechanisms from walking distance were not examined in this project. However, several studies have found that low-intensity exercise such as walking is strongly associated with lower blood pressure, lower insulin resistance, increased levels of HDL cholesterol, more efficient glucose metabolism and reduced levels of body fat [4,5]. These beneficial effects on biological mechanisms may consequently prevent the metabolic syndrome [33] and decrease developing CVD [34,35]. Physical activity may also bring high quality of life and well-being for elderly people [36].

In this study population, 17% had a history of diabetes. Most of them were taking oral hypoglycemic agents and only a small percentage of participants were taking insulin (2.5%) [12]. Therefore, we did not consider the functional role of insulin therapy in the outcome, although insulin therapy might reduce the risk of CVD in elderly people [37,38].

The reduction in the risk of CHD among individuals with diabetes is likely due to the beneficial effects of walking on glucose metabolism, including fasting and 2 h post load glucose levels [1,11]. This cohort has shown that both fasting and 2 h post load glucose levels are independently associated with CVD, after taking other established CVD risk factors into account [11]. Although the exact mechanism by which walking distance leads to a decrease in CHD incidence, independently of the risk factors we adjusted for, is not known, we hypothesize that walking leads to improved insulin sensitivity and better glucose control of both fasting and post load glucose. Furthermore, we estimate this process positively correlates with walking distance.

Given that walking is known to have significant health benefits in persons tolerant of moderate exercise, it is recommended to encourage older individuals with diabetes who are capable of physical activity to engage in walking on a daily basis with the approval of their physicians. The present study considered only men who are capable of physical activity. However, diabetes is a major cause of disability in the elderly population. They are also at high risk of CHD in the United States [39,40,41,42], therefore further research on the relationship between diabetes and disabilities is recommended. In addition, further data from clinical trials and intervention studies conducted in populations with diabetes would provide evidence to confirm these very encouraging findings for the prevention of CHD in older people with diabetes.

## 5. Conclusions

These findings suggest that among men with diabetes who are capable of physical activity, walking reduces CHD risk to levels similar to those of men without diabetes. Walking is an easy, safe, accessible, feasible and inexpensive form of physical activity for all ages and this investigation indicates it is especially useful among individuals with diabetes.

## Figures and Tables

**Table 1:** Age-Adjusted Incidence rate, 1000 person-years of Coronary Heart Disease (CHD) Based on 5 to 7 years of Follow-Up According to Walking Distance in Elderly Men Aged 71 to 93 years among men with diabetes and without diabetes.

Walking Distance, miles/day	Number of Subjects (%)		Number of Events (%)		Age-Adjusted Incidence Rate, per 1000 Person-Years (95% CI)	
	With Diabetes	Without Diabetes	With Diabetes	Without Diabetes	With Diabetes	Without Diabetes
<0.25	161 (30)	633 (30)	23 (38)	44 (30)	27.1 (24.6–29.6) *	12.7 (11.6–13.9) *
0.25 to 1.5	220 (41)	831(39)	27 (44)	69 (46)	23.2 (21.2–25.1) *	13.9 (12.3–15.0) *
>1.5	157 (29)	645 (31)	11(18)	36 (24)	12.2 (4.7–19.6)	9.1 (6.0–12.0)

* Significant difference between men with diabetes and without diabetes.

**Table 2:** Age-Adjusted Mean Levels (SD) and percent of Selected Risk Factors for CHD at Time of Baseline Examination (1991–1993) According to Walking Distance per Day among men with diabetes and without diabetes.

Baseline (1991–1993) Risk Factor	Walking Distance, miles/day					
	<0.25 miles/day		0.25 to 1.5 miles/day		>1.5 miles/day	
	With Diabetes	Without Diabetes	With Diabetes	Without Diabetes	With Diabetes	Without Diabetes
Age	77.4 (4.6)	77.60 (4.3) ^‡^	77.2 (3.9) ^†^	77.6 (4.3) ^§^	76.2 (3.5)	76.6 (3.9)
BMI	24.0 (3.4)	23.2 (3.3)	23.8 (3.3)	23.3 (3.0)	24.1 (2.8)	23.5 (2.9)
Fasting glucose, mg/dL	144.9 (42.1)	103.0 (9.1)	145.5 (45.2)	102.7 (8.4)	146.9 (45.4)	103.5 (8.6)
Total cholesterol, mg/dL	190.4 (190.4)	191.5 (34.2)	190.6 (34.9)	190.4 (31.8)	193.5 (32.4)	194.8 (31.4)
HDL cholesterol, mg/dL	48.3 (11.0)	53.3 (13.8)	49.2 (14.9)	52.1 (13.6)	48.6 (12.4)	52.6 (12.8)
SBP, mmHg	153.0 (24.5)	148.9 (23.2) ^†^	151.3 (22.7)	149.4 (22.0)	154.6 (23.7)	150.9 (22.4)
Current smoker, %	7.8 ^†^	11.3 ^‡^	5.90	7.30	2.50	5.40
Alcohol intake, oz/month	21.4 (45.2)	20.3 (40.1)	25.1 (57.6)	20.3 (41.6)	13.7 (26.0)	17.1 (33.7)

^†^: *p* < 0.05; ^‡^: *p* < 0.01; ^§^: *p* < 0.001: significant difference from men who walked >1.5 miles/day.

**Table 3:** Estimated Age-Adjusted and Risk Factor-Adjusted Relative Risks of CHD Comparing Ranges of Walking Distance per Day among men with diabetes and without diabetes.

Walking Distance, miles/day	Age-Adjusted Relative Risk (95% CI)		Risk Factor-Adjusted Relative Risk (95% Cl) *	
	With Diabetes	Without Diabetes	With Diabetes	Without Diabetes
<0.25 ^†^	1	1	1	1
0.25 to 1.5	0.77 (0.45–1.32)	1.07 (0.75–1.54)	0.81 (0.47–1.41)	1.16 (0.78–1.72)
>1.5	0.48 (0.24–0.98) ^‡^	0.70 (0.46–1.07)	0.45 (0.22–0.93) ^‡^	0.74 (0.47–1.17)
test for trend ^§^	*p* = 0.043	*p* = 0.070	*p* = 0.025	*p* = 0.10

* RRs are adjusted for age, BMI, SBP, fasting glucose, total and HDL cholesterol, alcohol consumption, and smoking; ^†^ Used the lowest level of walking (<0.25) as a reference group against higher levels of walking; ^‡^ Significant excess of CHD (*p* < 0.05); ^§^ Walking distance was treated as a continuous variable in test for trend.
